# Sunnyside

**Published:** 1881-07

**Authors:** 


Sunnyside.—Any of our Southern friends visiting New York, either for business or pleasure, would find an hour or so spent at this delightful "Private Medical Home for Nervous Invalids," a very pleasant change from the dust and heat of the city; and Dr. Edward C. Mann, the gentlemanly and intelligent medical superintendent would take great pleasure in showing them the grounds, buildings, etc., and rendering such a visit an agreeable one in every respect. His city office is at 28 West Thirtieth street, New York.

